# Mechanical effects of surgical procedures on osteochondral grafts elucidated by osmotic loading and real-time ultrasound

**DOI:** 10.1186/ar2801

**Published:** 2009-09-02

**Authors:** Koji Hattori, Kota Uematsu, Tomohiro Matsumoto, Hajime Ohgushi

**Affiliations:** 1 Research Institute for Cell Engineering, National Institute of Advanced Industrial Science and Technology, 3-11-46, Nakoji, Amagasaki, Hyogo 661-0974, Japan; 2 Department of Orthopaedic Surgery, Nara Medical University, 840, Shijyo-cho, Kashihara, Nara 634-8522, Japan

## Abstract

**Introduction:**

Osteochondral grafts have become popular for treating small, isolated and full-thickness cartilage lesions. It is recommended that a slightly oversized, rather than an exact-sized, osteochondral plug is transplanted to achieve a tight fit. Consequently, impacting forces are required to insert the osteochondral plug into the recipient site. However, it remains controversial whether these impacting forces affect the biomechanical condition of the grafted articular cartilage. The present study aimed to investigate the mechanical effects of osteochondral plug implantation using osmotic loading and real-time ultrasound.

**Methods:**

A full-thickness cylindrical osteochondral defect (diameter, 3.5 mm; depth, 5 mm) was created in the lateral lower quarter of the patella. Using graft-harvesting instruments, an osteochondral plug (diameter, 3.5 mm as exact-size or 4.5 mm as oversize; depth, 5 mm) was harvested from the lateral upper quarter of the patella and transplanted into the defect. Intact patella was used as a control. The samples were monitored by real-time ultrasound during sequential changes of the bathing solution from 0.15 M to 2 M saline (shrinkage phase) and back to 0.15 M saline (swelling phase). For cartilage sample assessment, three indices were selected, namely the change in amplitude from the cartilage surface (amplitude recovery rate: ARR) and the maximum echo shifts from the cartilage surface and the cartilage-bone interface.

**Results:**

The ARR is closely related to the cartilage surface integrity, while the echo shifts from the cartilage surface and the cartilage-bone interface are closely related to tissue deformation and NaCl diffusion, respectively. The ARR values of the oversized plugs were significantly lower than those of the control and exact-sized plugs. Regarding the maximum echo shifts from the cartilage surface and the cartilage-bone interface, no significant differences were observed among the three groups.

**Conclusions:**

These findings demonstrated that osmotic loading and real-time ultrasound were able to assess the mechanical condition of cartilage plugs after osteochondral grafting. In particular, the ARR was able to detect damage to the superficial collagen network in a non-destructive manner. Therefore, osmotic loading and real-time ultrasound are promising as minimally invasive methods for evaluating cartilage damage in the superficial zone after trauma or impact loading for osteochondral grafting.

## Introduction

Osteochondral grafts have become popular for the treatment of small, isolated and full-thickness cartilage lesions [1]. Osteochondral grafts have several advantages, including a high survival rate of the grafted articular cartilage, reliable bone union and no threat of disease transmission [1-3]. Several osteochondral transplantation systems are commercially available in clinical practice. For most of these systems, it is recommended that a slightly oversized, rather than an exact-sized, osteochondral plug is transplanted to achieve a tight fit [4], because plug stability is an important factor for optimal in-growth of a transplanted plug [5]. Therefore, impacting forces are required to insert the osteochondral plug into the recipient site during the osteochondral grafting procedure.

It remains controversial whether the impacting forces required to insert an osteochondral plug affect the biomechanical condition of the grafted articular cartilage. We previously developed an ultrasonic evaluation system for articular cartilage. We demonstrated that this system can be used to quantitatively clinically evaluate cartilage degeneration [6,7]. Using the same ultrasonic evaluation system, Kuroki and colleagues [8] examined the mechanical effects of the osteochondral grafting procedure on porcine articular cartilage immediately after surgery. The study indicated that osteochondral graft surgery does not affect the stiffness, surface irregularity or thickness of either oversized and exact-sized plugs. In contrast, Nishitani and colleagues [9] assessed osteochondral grafting of the human elbow using this system and showed that the cartilage plug may become damaged during the osteochondral grafting procedure. Nakaji and colleagues [10] evaluated the mechanical properties of cartilage plugs using a tactile sensor system and showed that the stiffness of oversized cartilage plugs did not differ significantly from that of the normal cartilage immediately after surgery. However, it is well known that the impacting forces required to implant an osteochondral graft can lead to chondrocyte death and fissure formation in the surface of the cartilage plug [11,12]. Therefore, it is speculated that the above described evaluation methods are not suitable for the assessment of articular cartilage damage from the impacting forces used to implant an osteochondral graft. Therefore, a more adjustable measurement method is required.

Ultrasound was first used to measure the osmotic swelling of articular cartilage by Tepic and colleagues [13]. Further studies have recently been carried out by Zheng and colleagues [14] and Wang and colleagues [15,16], who developed a new ultrasound system for monitoring transient depth-dependent osmotic swelling and solute diffusion in articular cartilage. Using this system, they successfully monitored articular cartilage digestion by trypsin in real time. Ultrasound assessment by osmotic loading can provide transient and depth-dependent swelling information for articular cartilage *in situ*. Therefore, osmotic loading and real-time ultrasound have the potential for assessing the cartilage damage caused by the impacting forces required to insert a plug during the osteochondral graft procedure. However, it remains unknown whether osmotic loading and real-time ultrasound can assess the mechanical condition of a cartilage plug after osteochondral grafting.

The purpose of the present study was to evaluate the mechanical effects of osteochondral plug implantation using osmotic loading and real-time ultrasound and to demonstrate the accuracy of ultrasound in identifying the cartilage damage after osteochondral graft procedures. To this end, we evaluated oversized and exact-sized cartilage plugs after osteochondral grafting. In the present study, we also assessed the cartilage plugs using a conventional mechanical test and observed the cartilage surface morphology by scanning electron microscopy (SEM).

## Materials and methods

### Cartilage sample processing

Porcine knee joints (n = 30) with intact capsules and ligaments were purchased from a slaughterhouse. After removal of the soft tissues, the knee joints were opened. The patellas with visually intact surfaces were harvested, wrapped in wet gauze soaked with physiological saline solution and stored at -20°C until use. For sample preparation, each patella was thawed at room temperature for one hour and immersed in physiological saline solution (0.15 M sodium chloride (NaCl)), before the lateral lower and upper quarters of the patella were cut using a band saw (K-100; Hozan Tool Industrial Co. Ltd., Osaka, Japan). During the processing steps described below, the cartilage surface was kept moist with physiological saline solution without immersing the sample.

**Figure 1:**
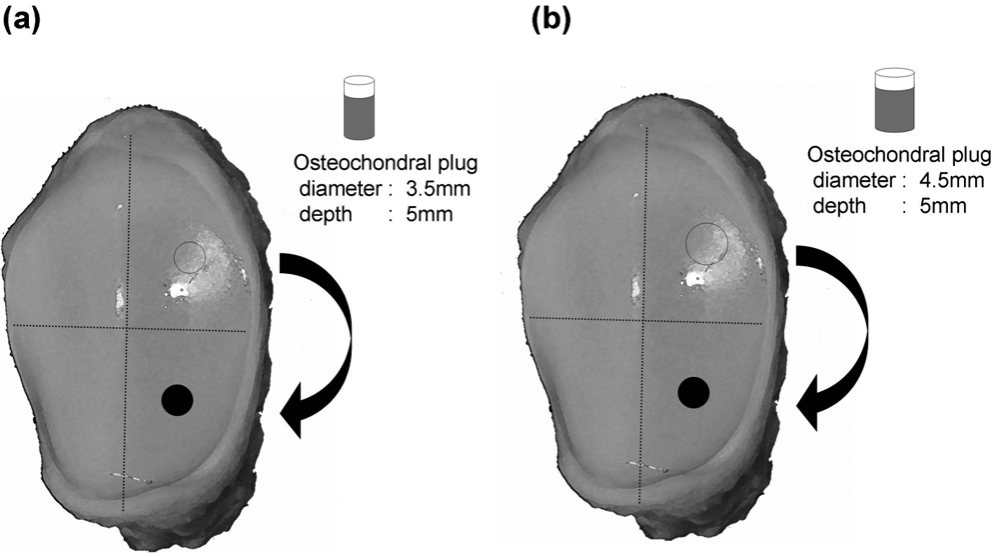
Sample preparation. A full-thickness osteochondral defect (closed circle; diameter, 3.5 mm; depth, 5 mm) is created in the lateral lower quarter of each patella. **(a) **Group I. An exact-sized plug (open circle) is harvested from the lateral upper quarter of the patella and transplanted into the defect. **(b) **Group II. An oversized plug (open circle) is harvested from the lateral upper quarter of the patella and transplanted into the defect.

A full-thickness cylindrical osteochondral defect (diameter, 3.5 mm; depth, 5 mm) was created in the lateral lower quarter of the patella. Using graft-harvesting instruments (MOSAICPLASTY System; Smith & Nephew Inc., Andover, MA, USA), an osteochondral plug (diameter, 3.5 or 4.5 mm; depth, 5 mm) was harvested from the lateral upper quarter of the patella. The samples were divided into two groups based on the surgical procedure (Figures 1a, b). In group I (n = 10), an exact-sized plug (diameter, 3.5 mm; depth, 5 mm) was harvested and implanted into the osteochondral defect in the lower quarter of the patella. The osteochondral plug exactly matched the size of the defect and was easily inserted with an adjustable plunger so it was as flush as possible with the surrounding cartilage. In group II (n = 10), an oversized plug (diameter, 4.5 mm; depth, 5 mm) was harvested and implanted into the osteochondral defect in the lower quarter of the patella. The oversized plug was inserted into the defect in a press-fit manner. The plug was advanced using a delivery tamp and seated as flush as possible with the surrounding cartilage. All of the surgical procedures were performed by a specialist in knee surgery (KU). In the control group (n = 10), intact cartilage in the lower quarter of the patella was used.

### Ultrasound monitoring system

**Figure 2:**
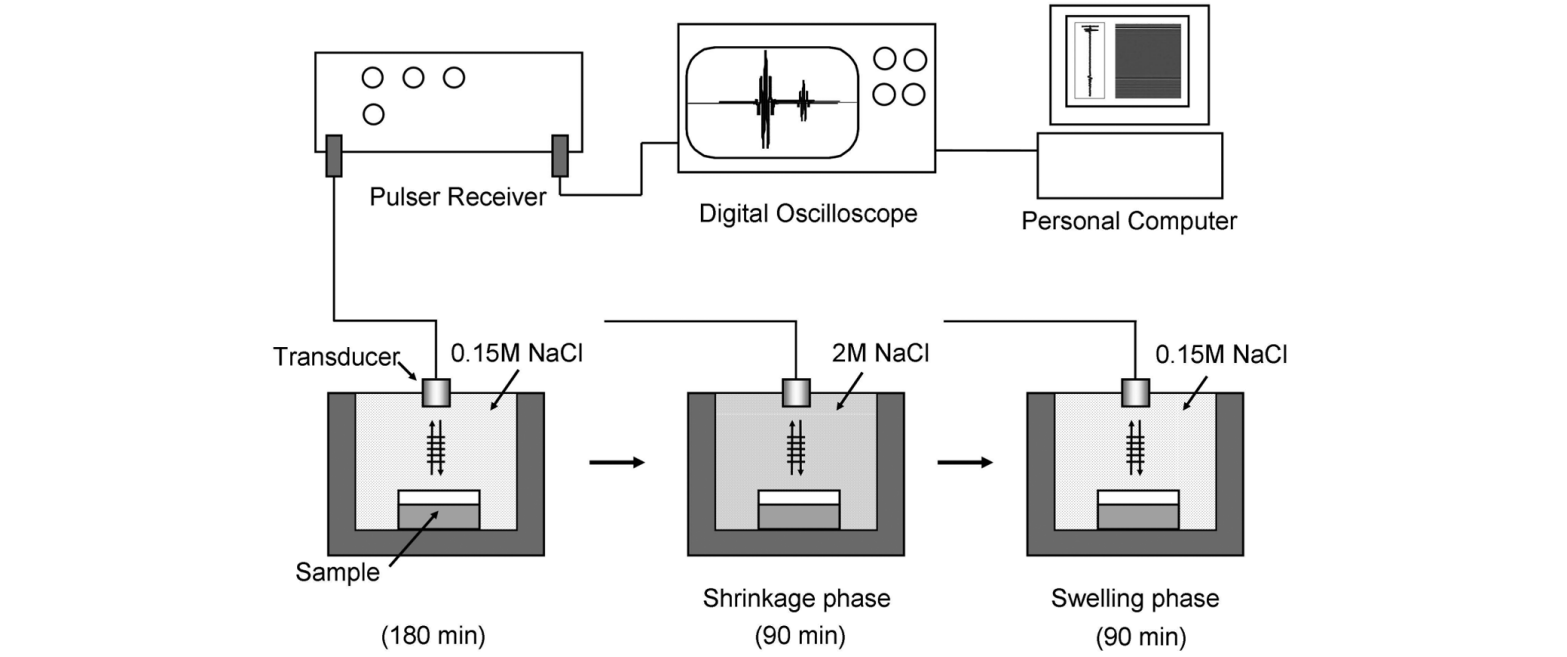
Schematic illustration of the osmotic loading and ultrasound monitoring system. The sample is fixed on the bottom of the container. NaCl = sodium chloride.

The ultrasound monitoring system used in this study was originally developed by Zheng and colleagues [14-16] and modified to a 10 MHz ultrasound system. The system was developed to monitor articular cartilage in terms of the transient depth-dependent swelling behaviour and the transport of solutes induced by changing the concentration of the bathing saline solution. A schematic outline of the ultrasound swelling measurement system is shown in Figure 2. The system included a 10 MHz transducer (diameter, 3 mm; thickness, 3 mm; flat ultrasonic wave), an ultrasonic pulser/receiver (Model 5800PR; Olympus NDT, Waltham, MA, USA), a digital oscilloscope (TDS 2022B; Tektronix Japan, Ltd., Tokyo, Japan) and custom-made software (LabVIEW 8.5; National Instruments, Austin, TX, USA) for data collection and signal processing.

### Ultrasound analysis

**Figure 3:**
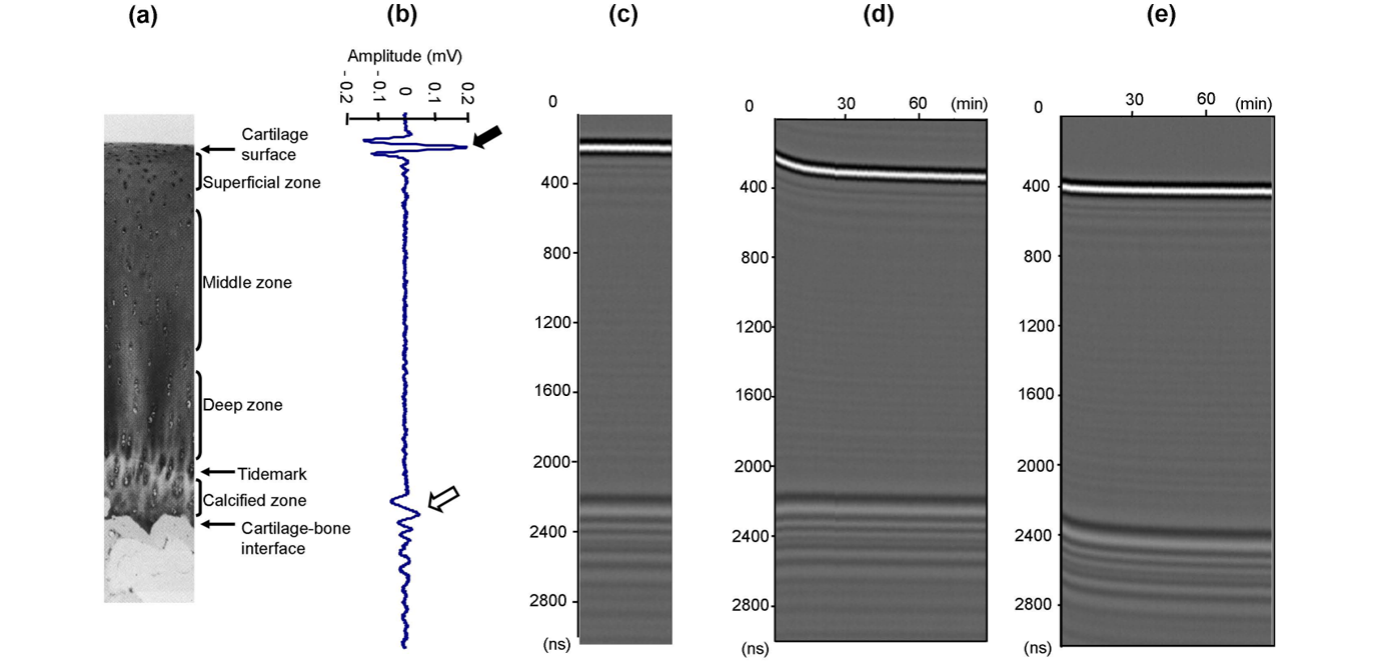
Imaging data from the osmotic loading and real-time ultrasound system. **(a) **Histology of a typical articular cartilage sample. **(b) **A-mode echogram from an articular cartilage sample. The black arrow indicates the amplitude from the cartilage surface and the white arrow indicates the amplitude from the cartilage-bone interface. The amplitude recovery rate was calculated from the change in the cartilage surface amplitude from the shrinkage phase to the swelling phase. **(c) **M-mode image before osmotic loading. The gray levels indicate the amplitudes of the ultrasound signals. **(d) **Typical M-mode image in the shrinkage phase. **(e) **Typical M-mode image in the swelling phase.

Each articular cartilage sample was placed on the bottom of the container and submerged in 0.15 M saline solution for three hours. The transducer was moved to a position perpendicularly above the cartilage surface of the osteochondral graft. After the three-hour immersion, the 0.15 M saline solution was rapidly removed from the container using a syringe and replaced with 2 M saline solution within 30 seconds, and the sample was monitored by ultrasound for 90 minutes (shrinkage phase). Subsequently, the 2 M saline solution was changed back to 0.15 M saline solution within 30 seconds, and the sample was monitored by ultrasound for 90 minutes (swelling phase). The echo signals that were reflected from the cartilage surface and the cartilage-bone interface and became scattered inside the articular cartilage layer were continuously recorded with a sampling period of 30 seconds (Figures 3a, b). The ultrasound signals were also displayed in M-mode images, with grey levels indicating the amplitudes of the ultrasound signals (Figures 3c to 3e). Horizontal traces of the cartilage surface in the M-mode images indicated the transient displacement (shrinkage/swelling) of the samples, while similar traces of the cartilage-bone interface indicated the diffusivity of the saline solution in the cartilage. All of the experiments were carried out at room temperature.

For cartilage sample assessment, we focused on three ultrasound indices, namely the change in amplitude from the cartilage surface and the echo shifts from the cartilage surface and the cartilage-bone interface. The change in amplitude from the cartilage surface refers to the change of the cartilage/saline solution acoustic impedance. In the shrinkage phase, cartilage is sufficiently dehydrated to relax the collagen network in the collagen-rich superficial zone. In the swelling phase, the impedance and amplitude increase as the proteoglycans swell, thereby stretching the collagen and increasing the stiffness [13]. Therefore, as one quantitative index of the cartilage assessment in this study, the amplitude recovery rate (ARR) was determined. The ARR value was expressed using the following equation:

- where MAMP swelling is the mean amplitude from the cartilage surface in the swelling phase, and MAMP shrinkage is the mean amplitude from the cartilage surface in the shrinkage phase.

We also evaluated the echo shifts from the cartilage surface and the cartilage-bone interface in both the shrinkage and swelling phases. The echo shift from the cartilage surface indicates the sample displacement, while the echo shift from the cartilage-bone interface indicates the diffusivity of the saline solution in the sample [14]. Therefore, as the other quantitative indices of the cartilage assessment in this study, the maximum echo shifts were chosen.

### Morphological analysis

Two samples in each group were subjected to morphological analysis using an SEM (Model SM-350; Topcon Technohouse Corporation, Tokyo, Japan). The samples were fixed in 2% glutaraldehyde buffered with 0.1 M cacodylate, dehydrated in a graded ethanol series, dried using the critical point technique and coated by sputtering with a gold layer [17].

### Biomechanical analysis

Eight cartilage samples were immersed in physiological saline and tested within three hours. To determine the mechanical properties of the grafted cartilage, an electromechanical material testing machine (EZ-L; Shimadzu Corporation, Kyoto, Japan) was used. Forces were applied to the grafted cartilage at a displacement rate of 2.0 mm/min using a 3.0 mm diameter solid aluminum indenter. A load-deformation curve was obtained during the compression. As biomechanical parameters, we defined the maximum load (breaking load: F max) applied at fracture of the grafted cartilage.

### Statistical analysis

For multiple comparisons of ultrasound findings, the groups were analyzed using the nonparametric Kruskal-Wallis test. When significant variance was detected, the differences among individual groups were determined using the Mann-Whitney U test with the Bonferroni correction. For comparisons between two groups in the biomechanics analyses, the differences were analyzed by the nonparametric Mann-Whitney U test. The significance level was set at *P *< 0.05.

## Results

### Ultrasonic findings

**Figure 4:**
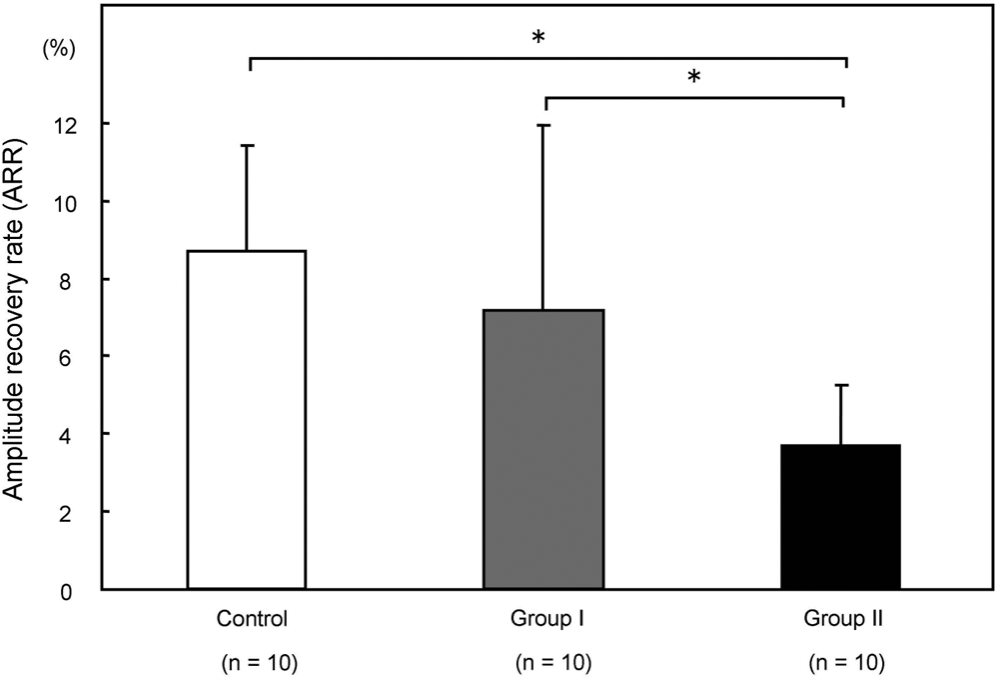
Mean amplitude recovery rate values of the three groups. The error bars represent the standard deviation of each group. *P < 0.05 by the nonparametric Kruskal-Wallis test.

The ARR values (mean ± standard deviation) were 8.64 ± 2.70% in the control group, 7.14 ± 4.74% in group I and 3.41 ± 1.58% in group II (Figure 4). A significant difference in the ARR was observed between the control group and group II (*P *= 0.008) and between group I and II (*P *= 0.024).

**Figure 5:**
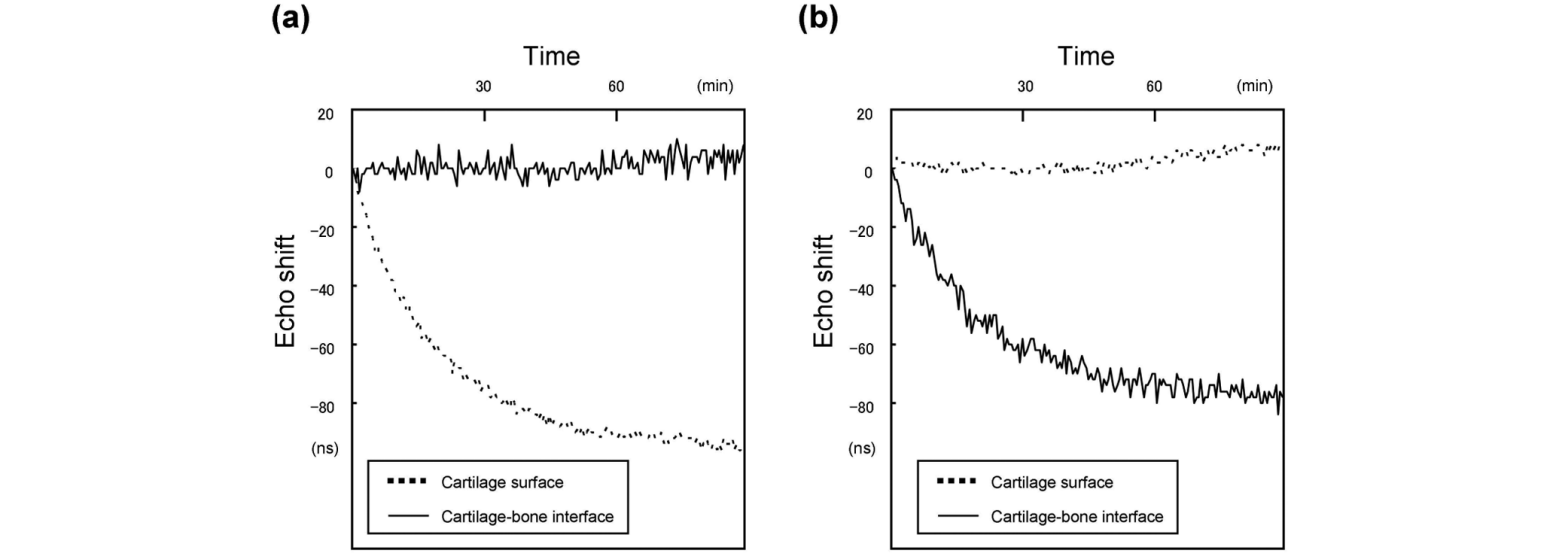
Time courses of echo shifts. **(a, b) **Time courses of the echo shifts from the cartilage surface (dotted line) and the cartilage-bone interface (thick line) in the **(a) **shrinkage phase and **(b) **swelling phase.

Figure 5 shows the typical time courses of the echo shifts of the control cartilage in the shrinkage phase (Figure 5a) and swelling phase (Figure 5b). The patterns of the echo shifts were similar in all three groups. There was a rapid decrease in the echo shift from the cartilage surface after 30 minutes of immersion in 2 M NaCl (shrinkage phase), followed by a gradual decrease from 30 to 90 minutes. There was a rapid decrease in the echo shift from the cartilage-bone interface after 30 minutes of immersion in 0.15 M NaCl (swelling phase), followed by a gradual decrease from 30 to 90 minutes. The maximum echo shifts are shown in Table 1. There were no significant differences in the maximum echo shifts among the three groups.

**Table 1:** Echo shifts from cartilage surface and cartilage-bone interface in the shrinkage and swelling phases

	Control (n = 10)	Group I (n = 10)	Group II (n = 10)	*P *value
Shrinkage phase				
Cartilage surface	-82.6 ± 26.1 ns	-77.4 ± 22.7 ns	-70.7 ± 27.8 ns	NS
Cartilage-bone interface	5.2 ± 24.8 ns	14.2 ± 24.8 ns	22.4 ± 16.6 ns	NS
Swelling phase				
Cartilage surface	-9.2 ± 21.5 ns	-2.6 ± 15.0 ns	4.4 ± 9.6 ns	NS
Cartilage-bone interface	-86.0 ± 18.1 ns	-74.8 ± 12.9 ns	-69.1 ± 19.2 ns	NS

Data are presented as mean ± standard deviation. *P *value based on Kruskal-Wallis test. The significance level was set at *P *< 0.05. NS = not significant.

### Morphological findings

**Figure 6:**
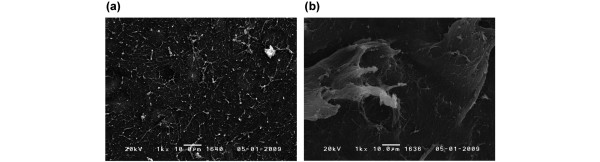
Representative cartilage surface images obtained by scanning electron microscopy. **(a) **Articular surface of a cartilage plug in group I. **(b) **Articular surface of a cartilage plug in group II.

Representative SEM images from samples in groups I and II are shown in Figure 6. In group I, there were tiny irregularities in the surface of the cartilage plug. However, the superficial collagen network was not ruptured (Figure 6a). In contrast, most of the cartilage surface in group II was damaged by the surgical processing. The superficial collagen network was broken and the cartilage superficial layer had partially peeled away (Figure 6b).

### Biomechanical findings

**Figure 7:**
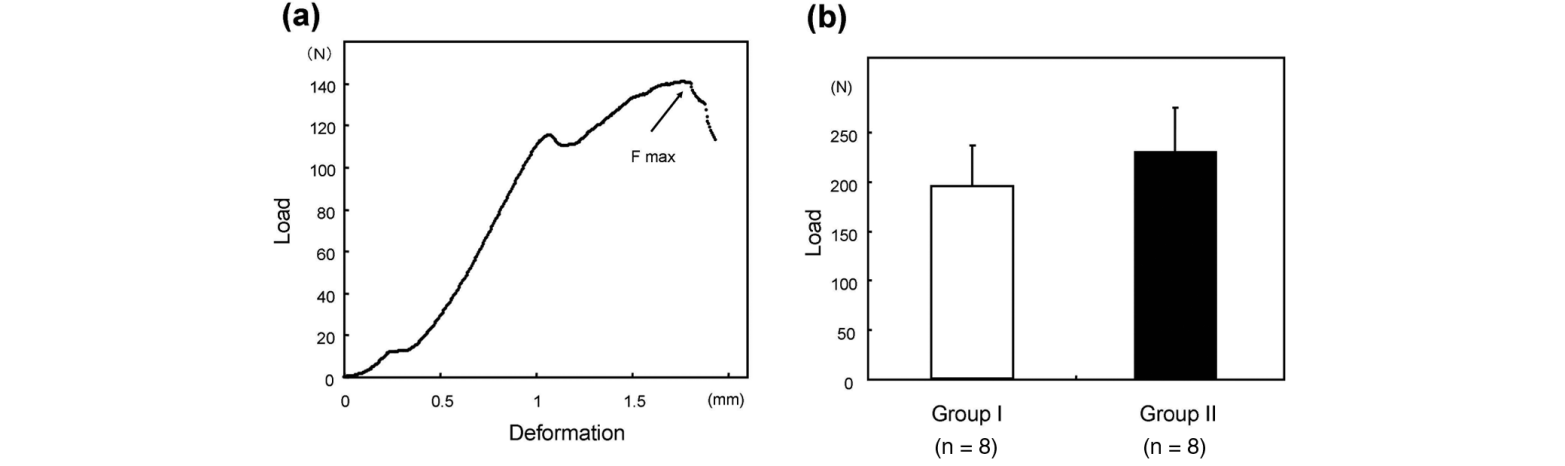
Biomechanical analysis. **(a) **Load-deformation curve of the sample. The maximum load applied at fracture of the sample (breaking load) is shown as F max.**(b) **Breaking loads (F max) of groups I and II. The error bars represent the standard deviation of each group. *P *< 0.05 by the nonparametric Mann-Whitney U test.

A load-deformation curve is shown in Figure 7a. The F max values were 198.1 ± 42.2 N in group I and 233.2 ± 46.2 N in group II (Figure 7b). The mean F max value was higher in group II than in group I, but the difference was not significant (*P *= 0.14).

## Discussion

The present study investigated the osmotic shrinkage-swelling behaviours of oversized and exact-sized cartilage plugs in osteochondral grafting using osmotic loading and real-time ultrasound. The main findings of the study are that osmotic loading and real-time ultrasound are capable of assessing the mechanical condition of a cartilage plug after osteochondral grafting. In particular, the ARR was able to detect damage to the superficial collagen network in a non-destructive manner. Therefore, osmotic loading and real-time ultrasound are promising as minimally invasive methods for evaluating cartilage damage in the superficial zone after trauma or impact loading for osteochondral grafting.

An osteochondral plug that is exactly the same size and shape as a cartilage defect seems to be ideal for osteochondral grafting. However, Makino and colleagues [18] reported that histological changes occur in the implanted cartilage, after examining osteochondral grafts taken from the femoral condyle and returned to their original sites. In their rabbit model, the graft was not strictly the same size as the defect because of the blade thickness of the chisel used to take the graft. Moreover, they revealed that an oversized osteochondral graft appeared to be almost the same as the normal adjacent cartilage at 4, 12 and 24 weeks after surgery [4]. Therefore, an oversized plug can be recommended for use in the osteochondral graft procedure. However, the impact load required to insert a plug into the recipient site is higher for an oversized plug than for an exact-sized plug.

Impact loading of articular cartilage has commonly been associated with structural damage [19-22], loss of viability and changes in the metabolism of chondrocytes [19,22-24], with subsequent degeneration of the articular cartilage [25]. In general, evaluations of damage to cartilage have been performed by histological analysis of the structural integrity [19,22], SEM imaging of the surface morphology [17], assessment of tissue swelling by the water content related to disruption of collagen fibrils [19,23], assessment of chondrocyte death [19,24] and release of cartilage macromolecular constituents during subsequent tissue culture [19,22,24]. However, these analyses require the collection of cartilage tissue samples, which will result in damage to the cartilage plug surface. Therefore, all the above described evaluation methods should be avoided in clinical practice.

There are several imaging modalities to assess articular cartilage such as radiograph, computed tomography (CT), magnetic resonance imaging (MRI) and optical coherence tomography (OCT). Radiograph and CT do not image soft tissue, which prevent identification of structural changes of articular cartilage. Conventional MRI has been used in clinical practice to measure morphological change in articular cartilage. In comparison with MRI, the present ultrasonic approach may allow real-time monitoring of depth-dependent osmotic behaviours by the echo shift and the changes in amplitude. Moreover, the present system is much less expensive in comparison with MRI. OCT is a novel form of optical imaging that enables cross-sectional visualization of tissue micro architecture. However, OCT is still in its early stages of development for the assessment of articular cartilage [26,27]. Therefore, further studies to assess articular cartilage from the view point of biomechanics are required.

Tepic and colleagues [13] developed an ultrasonic system for assessing osmotic swelling of articular cartilage after dehydration in humid air. However, their ultrasonic system was only able to evaluate the whole cartilage layer and no measurements were obtained for depth-dependent swelling behaviours. Zheng and colleagues developed a new ultrasound system for monitoring transient depth-dependent osmotic swelling and solute diffusion in articular cartilage [14-16]. Consequently, osmotic loading and real-time ultrasound can provide comprehensive information about the biomechanical behaviour of articular cartilage. The present study has demonstrated the feasibility of this system for evaluating cartilage damage caused by impact loading while inserting a plug during the osteochondral graft procedure.

In this study, cartilage plugs were assessed not only by their osmotic shrinking and swelling behaviours but also by the changes in amplitude of the cartilage surface from the shrinkage phase to the swelling phase. A previous study revealed that the amplitude from the cartilage surface is related to the tissue reflection coefficient, acoustic impedance, elastic modulus and surface condition in physics, and related to proteoglycan depletion and collagen disruption in biology [28-30]. In the present study, the cartilage plugs were damaged by the impact loading required for their insertion into the defects. Moreover, damage to the surface collagen network was confirmed by SEM. By using osmotic swelling, differences in the cartilage surface integrity between oversized cartilage plugs and intact cartilage were enhanced. As a result, the ARR of oversized cartilage plugs was significantly lower than that of intact cartilage. Therefore, the ARR mainly reveals the microstructural changes to the articular cartilage in the superficial collagen-rich zone.

On the other hand, the echo shift from the cartilage surface is known to reflect the sample displacement and the echo shift from the cartilage-bone interface is known to reflect the diffusivity of saline solution in the sample [14]. In the present study, the echo shifts of oversized and exact-sized cartilage plugs were similar to those of intact cartilage. These results suggest that the interiors of the cartilage plugs were not damaged by the impact loading required to insert the plugs into the defects. Within the limitations of the measurement accuracy, the mechanical indentation test could not detect damage to the cartilage surface. Therefore, osmotic loading and real-time ultrasound represent new approaches for studying the biomechanical and biophysical aspects associated with articular cartilage.

Three limitations of our study should be considered. First, we did not examine the effects of osmotic loading on the viability and metabolism of chondrocytes. A high concentration of NaCl may be harmful to cartilage tissues. If this proves to be the case, the methodology for the osmotic loading should be changed from 2 M and 0.15 M NaCl to humid air and 0.15 M NaCl [13]. Second, the impact loading required to insert the osteochondral plugs could not be controlled. However, the present study simulated an assessment of human osteochondral grafts, and a surgeon who was experienced in the osteochondral grafting procedure performed the harvesting and implantation procedures. Therefore, damage to the collagen network in the superficial layer of cartilage plugs would occur during the osteochondral grafting procedure.

Finally, the present study was carried out to investigate the feasibility of using osmotic loading and real-time ultrasound to assess the shrinking and swelling behaviors of cartilage plugs after osteochondral grafting. If the present study design were applied to clinical practice, the length of measurement time would come into question. However, maximum deformation of ARR and echo shift in plug cartilage by changing the saline concentration occurred during the first several minutes [14]. Thus, with proper miniaturization of the design, it would be clinically practical to detect cartilage damage after the osteochondal graft procedure. Therefore, for application to clinical situations, further studies are required to determine whether this system will prove beneficial for the assessment of human osteochondral grafts.

## Conclusions

The present study has obtained the first data for the assessment of articular cartilage damage caused by the impact loading required to insert an osteochondral plug using osmotic loading and real-time ultrasound. Under osmotic loading, the changes in the amplitude and echo shifts can support the evaluation of cartilage damage in osteochondral grafts. Moreover, osmotic loading and real-time ultrasound may contribute to tissue engineering in the musculoskeletal field, and the ARR and echo shifts can be expected to become quantitative indices for the biomechanical and biophysical properties of articular cartilage.

## Abbreviations

ARR: amplitude recovery rate; CT: computed tomography; MRI: magnetic resonance imaging; NaCl: sodium chloride; ORT: optical coherence tomography; SEM: scanning electron microscopy.

## Competing interests

The authors declare that they have no competing interests.

## Authors' contributions

KH conceived the study, participated in its design and performed all the experiments. KU performed the harvesting and implantation procedures of the cartilage samples. TM performed the SEM assessments. HO participated in the study design and the biomechanical analyses. All authors have read and approved the final manuscript.
